# Marine Prasinoviruses and Their Tiny Plankton Hosts: A Review

**DOI:** 10.3390/v9030043

**Published:** 2017-03-15

**Authors:** Karen D. Weynberg, Michael J. Allen, William H. Wilson

**Affiliations:** 1 Australian Institute of Marine Science, PMB 3, Townsville, Queensland 4810, Australia; 2 Plymouth Marine Laboratory, Prospect Place, Plymouth PL1 3DH, UK; mija@pml.ac.uk; 3 Sir Alister Hardy Foundation for Ocean Science, The Laboratory, Citadel Hill, Plymouth PL1 2PB, UK; wilwil@sahfos.ac.uk

**Keywords:** virus–host interactions, marine virus ecology, virus-driven evolution

## Abstract

Viruses play a crucial role in the marine environment, promoting nutrient recycling and biogeochemical cycling and driving evolutionary processes. Tiny marine phytoplankton called prasinophytes are ubiquitous and significant contributors to global primary production and biomass. A number of viruses (known as prasinoviruses) that infect these important primary producers have been isolated and characterised over the past decade. Here we review the current body of knowledge about prasinoviruses and their interactions with their algal hosts. Several genes, including those encoding for glycosyltransferases, methyltransferases and amino acid synthesis enzymes, which have never been identified in viruses of eukaryotes previously, have been detected in prasinovirus genomes. The host organisms are also intriguing; most recently, an immunity chromosome used by a prasinophyte in response to viral infection was discovered. In light of such recent, novel discoveries, we discuss why the cellular simplicity of prasinophytes makes for appealing model host organism–virus systems to facilitate focused and detailed investigations into the dynamics of marine viruses and their intimate associations with host species. We encourage the adoption of the prasinophyte *Ostreococcus* and its associated viruses as a model host–virus system for examination of cellular and molecular processes in the marine environment.

## 1. Introduction

Viruses are the most diverse and abundant biological entities in the world’s oceans, with estimates often reaching in excess of 10^8^ viruses per millilitre (mL) of seawater [1,2]. The advent of techniques such as epifluorescence microscopy and flow cytometry initially helped to reveal the sheer abundance of viruses in the world’s ocean, and allowed the ‘viral shunt’ to be incorporated into the classic food web scenario [3,4,5,6]. Building on these observations, isolation, molecular and physiological studies have shown that marine viruses exert a great influence on nutrient and energy cycling, biogeochemistry, population dynamics, genetic exchange and evolution in the marine environment [4,5].

Marine microbes comprise more than 90% of the living biomass in the global oceans [4,5,7] and the phytoplankton component of this biomass are responsible for approximately half of the world’s photosynthetic activity [8,9]. Phytoplankton have very high turnover rates, with the global phytoplankton population estimated to be replaced on average once every week [9]. Thus, phytoplankton have the potential to adapt to rapidly changing environmental conditions, which in light of current climate change predictions [10,11] will likely be of great significance. The simple cell structure and biology of these tiny ocean-dwelling autotrophs, coupled with rapidly increasing knowledge data on their associated viruses, has led to the establishment of new host–virus model systems [12]. Through infection, disruption and manipulation of cellular function and resulting cellular mortality, viruses influence the dynamics of key processes including primary production in the oceans. Host–virus model systems are proving increasingly important since classic oceanic food webs and nutrient cycling models have, until recently, tended to overlook the role of viruses. However, there are some notable exceptions, with recent marine microbial models now beginning to incorporate the role of viruses [13].

Over the last decade, the field of marine virology has widened to include studies on viruses that infect the smallest of the eukaryotic phytoplankton (typically less than 3 microns). Although the cyanobacteria *Synechococcus* and *Prochlorococcus* are two of the most productive genera in the open ocean [14,15,16], microalgae of the class Prasinophyceae, particularly of the family Mamiellaceae, and notably the genera *Ostreococcus*, *Micromonas* and *Bathycoccus*, are significant contributors in coastal and estuarine waters [8,17]. The class Prasinophyceae is viewed as the most basal in the green plant lineage and is believed to be the root from which all other green algae and land plants emerged [18]. Phylogenetic analyses have revealed Prasinophyceae is a paraphyletic early branching lineage of the Chlorophyta [19]. The non-monophyletic classification of this class of algae can complicate the study of their coevolution with viruses.

These microalgae are generically referred to as picophytoplankton based on their size range (<3 microns). The majority of viruses isolated and described that infect eukaryotic phytoplankton have been large dsDNA viruses assigned to the family *Phycodnaviridae*, comprised of five genera: *Prasinovirus*, *Chlorovirus*, *Phaeovirus*, *Raphidovirus*, *Coccolithovirus*. The *Phycodnaviridae* are believed to share a common evolutionary ancestor with other viral families, namely the *Asfarviridae*, *Poxviridae*, *Iridoviridae*, *Ascoviridae*, *Mimiviridae*, *Marseilleviridae*, *Megaviridae*, *Pithoviridae*, and *Pandoraviridae* [20,21,22]. This collective of virus families infect a diverse range of eukaryotic hosts and are grouped as the Nucleo-Cytoplasmic Large DNA Viruses (NCLDVs) [20,21,23,24]. The NCLDVs are characterised as possessing large dsDNA genomes of greater than 100 kilobases (kb) with replication predominately occurring solely in the cytoplasm or, in certain interactions, initiating transcription in the nucleus before completion of assembly in the cytoplasm. Previously, it was proposed that the NCLDVs form a new order of giant viruses, the ‘Megavirales’ [25], although, to date, this nomenclature has not yet been widely adopted.

Genome sequences are now available for the host prasinophyte picophytoplankton species *Ostreococcus tauri* [26,27], *Ostreococcus lucimarinus* [27], *Micromonas pusilla* [28] and *Bathycoccus prasinos* [29], as well as 12 whole prasinovirus genome sequences [30,31,32,33,34] (Table 1). Analysis of the NCLDVs present in microbial metagenomes collected during the TARA Oceans survey indicated that prasinoviruses were the most abundant NCLDVs, outnumbering the next most abundant members of the Megavirales, Mimivirus [35]. High abundances of prasinoviruses have also been described in other waters [36]. Clearly, the role of prasinoviruses in the natural environment is significant, and the study of their host interactions will provide insight on evolutionary and population dynamics, as well as intracellular physiological function when viruses rewire biochemical pathways as part of their manipulation during infection. For example, certain prasinoviruses have been identified as encoding novel genes never before reported in viruses e.g., a prasinoviral genome, OtV-2, encodes the only cytosolic, non-membrane-bound haem-binding protein cytochrome *b_5_* [37]. Despite the precise metabolic role still remaining elusive, the very identification of this gene in the genome of a virus, which infects a host with such a small physical structure, is intriguing. Indeed, it suggests that within smaller and simpler physiological cellular systems, such as *Ostreococcus*, organizational constraints associated with membrane and cellular function and compartmentalisation may not necessarily be as stringent as in more complex cells.

Over recent decades, our knowledge about the role of viruses in the marine environment has increased, with a growing number of virus–host systems isolated from the environment now established under laboratory conditions. Genome data arising from studies on such systems help inform investigations of key interactions, including horizontal gene transfer (HGT), between algal hosts and their viruses. A high degree of conservation has been reported between the genomes of prasinoviruses that infect all three genera [31], hereon referred to as OVs (*Ostreococcus* viruses—OtV refers to *Ostreococcus tauri* virus; OlV refers to *Ostreococcus lucimarinus* virus; OmV refers to *Ostreococcus mediterraneus* virus); MpVs (*Micromonas pusilla* viruses); BpVs (*Bathycoccus prasinos* viruses).

In light of their significant contribution and abundance in the marine environment, this review assesses what has been revealed in recent studies about prasinoviruses and their host interactions. We also argue for the global adoption of a Prasinovirus model host–virus system to help unveil more about the dynamics of these intimate and important associations.

## 2. Prasinophyte Host Genomes and Ecology

To date, six Prasinophyceae genomes, all within the order Mamiellales, have been sequenced. The sequenced genomes of the Mamilellophyceae show a gene-dense genome structure with minimal redundancy, indicating a simple organisation and highly reduced gene copy numbers, often down to just a single gene copy. This reflects positively in terms of these eukaryotic algae being suitable model host organisms for virus studies. Quirks in host metabolism of these species may also aid enquiries into host–virus metabolic mechanisms. A complex history of gene acquisition appears to have occurred in prasinophytes, which possess relatively small eukaryotic genomes with evidently high levels of HGT. Although sexual reproduction has not been reported in Mamelliophyceae strains, there are genes present in both *Ostreococcus* and *Micromonas* for meiosis control and it would appear that genetic recombination does occur between strains within the same clade [38]. Sequenced *Micromonas* genomes are more divergent than the *Ostreococcus* genomes [28] and *Ostreococcus tauri* has fewer than 8000 genes. The genetic distances between different clades within a genus of these prasinophytes revealed two *Ostreococcus* strains belonging to two clades actually only showed a divergence of less than 0.5% but approximately 25% divergence in amino-acid identity in their orthologous protein coding genes [34].

Two of the *Ostreococcus* host genomes that have been sequenced (*O. tauri* strain OTH95 [26] and *O. lucimarinus* strain CCE9901 [27]) have been described as high-light adapted coastal strains, whilst the *Ostreococcus* sp. strain RCC809 is viewed as a low-light adapted open-oceans species (genome available at [39,40]. Similar to high-light and low-light ecotypes seen in the cyanobacterium genus *Prochlorococcus* [41,42], there is evidence for niche adaptation within *Ostreococcus* [40], although further evidence is required to confirm this, as the occurrence of different *Ostreococcus* strains at various depths may be attributed to other factors, e.g., iron bioavailability or be indicative of acclimatisation, not adaptation. Three clades of *Ostreococcus*—A, C and D—are classified as high-light adapted, as defined through laboratory photophysiology assessments, and clade B is described as low-light adapted [40]. However, this low-light adapted clade has been reported in surface waters [8,43] and although different ecotypes do not commonly occur at the same geographical location, the drivers behind clade distribution may be more complex than determined by sea surface irradiance levels alone [43]. The putative low-light ecotype strain has increased photosensitivity and an adapted photoprotection mechanism.

*Ostreococcus* has recently been proposed as a suitable model organism for investigating iron uptake and metabolism in eukaryotic phytoplankton [44]. The bioavailability of iron in the oceans is known to be low, particularly in oligotrophic open ocean waters. The exact mechanisms involved in how marine microalgae manage to acquire iron are poorly understood. *O. tauri* has been shown to have tightly regulated diurnal cycling linked to iron uptake [44]. *Ostreococcus* sequenced genomes lack a suite of genes involved in iron transport with the exception of a multi-copper oxidase found only in *O. tauri* [27] indicating *Ostreococcus* uses an alternative iron acquisition pathway to other phytoplankton. A recent RNASeq analysis was conducted to examine cellular responses of *O. tauri* to a lack of iron over a diurnal cycle, making this the largest transcription study of *O. tauri* to date. Diurnal cycles were seen to be key to *O. tauri*’s metabolism of iron and via a non-reductive pathway, unlike the iron metabolism of *Chlamydomonas*. Instead of copper, zinc plays a major role in iron uptake in *O. tauri*, as many zinc finger proteins are involved in the upregulation of related genes. The mechanisms underpinning the utilisation of iron in this microalgal species differed greatly from those described for the established algal model *Chlamydomonas reinhardtii*. To date, no virus has been described that infects model species such as *Chlamydomonas* and yet we have an easy-to-study system in the unicellular *Ostreococcus* and its viruses that we can explore so much further. The findings that concluded that *Ostreococcus* has adopted an alternative iron uptake system to established model organisms, such as *Chlamydomonas,* add strength to the argument for establishment of this host system. Despite *C. reinhardtii* being a well-established model organism, there is a paucity of available technical tools, for example tightly regulated promoters and targeted gene inactivation and replacement, to study its cell cycle. *O. tauri* can undergo transformation [45] and its viruses could also be used as vectors and tools for manipulation. The complete chloroplast and mitochondrial genomes for *O. tauri* are also available to provide insights into processes such as photosynthesis [46]. The single chloroplast found in *Ostreococcus* cells is simple as it has a reduced size with just three layers of stacked thylakoid membranes.

## 3. *Micromonas* Viruses: The Roots of Algal Virology

**Figure 1:**
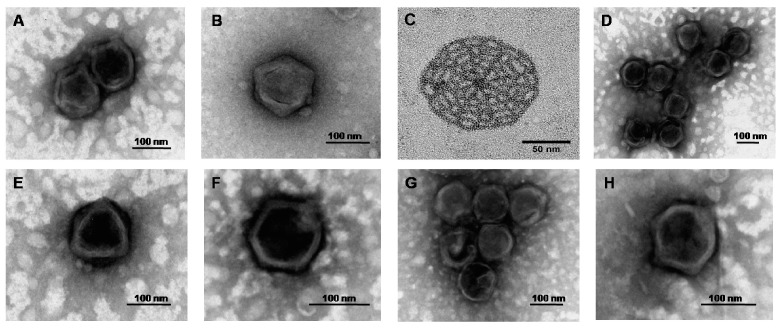
Negatively stained transmission electron microscopy micrographs of (**A**,**B**) *Micromonas pusilla* viruses (MpVs); (**C**) ‘Spiderweb’-like plate from exterior of *Bathycoccus prasinos* cell; (**D**–**H**) *O. tauri* viruses (OtVs).

The first report of phycodnavirus isolations was from the prasinophyte *Micromonas pusilla* [47]. These workers described the relatively easy method of isolating the virus from seawater and subsequent lysis of unialgal cultures. However, the results of this work were largely ignored at the time and no major investigations began until the high abundance of viruses in the marine environment was described by Bergh et al. in 1989 [1]. Until recently, *Micromonas*-specific viruses were the only viruses that infect prasinophytes to be described in any detail. Similar to other characterised prasinoviruses, transmission electron microscopy (TEM) reveals MpVs have icosahedral caspids with diameters ~120 nm consisting of electron-dense cores (Figure 1A,B). Most subsequent studies following the initial discovery of MpVs have largely investigated the ecological roles of these viruses and the genetic diversity they harbour [48,49,50,51,52,53]. It has been shown that *M. pusilla*-specific dsDNA viruses can lyse up to 25% of their host population on a daily basis [54]. The host species survives such high mortality rates through high growth rates and a high host diversity [8].

Transient blooms of *Micromonas* have been monitored and observed and *M. pusilla* has been reported as being the dominant member of the photosynthetic picoeukaryote assemblage all year round in certain locations, such as the Western English Channel, where a recent study revealed the dynamics of bloom-and-bust scenarios and the diversity of viral genotypes involved [55]. Whole genome sequences of two isolates of the *Micromonas* host species have been reported [28], which will help to unveil more about host–virus interactions. Somewhat surprisingly, for a long time only partial gene sequences, mainly of the DNA polymerase gene [56,57], were available in the public databases until Moreau and co-workers sequenced the complete genome of a dsDNA MpV [31] (Table 1).

## 4. *Bathycoccus* Viruses

The picophytoplankton genus *Bathycoccus* is comprised of a globally distributed green alga, which produces characteristic ‘spiderweb-like’ plates on the outside of the cells (Figure 1C). The whole genome sequence of the species *B. prasinos* was recently reported [29] and shows approximately 5% HGT, mainly from bacterial and eukaryotic origin with very little of viral origin [29]. Two of the 19 chromosomes found in *B. prasinos*, are described as outlier chromosomes with the small outlier chromosome (SOC) possibly playing a key role in the susceptibility of the alga to viral infection due to its observed hypervariability [29]. Interestingly, as will be discussed in detail, a SOC has been identified in *O. tauri* that has been shown to be key to providing resistance to viral infection [58]. In fact, outlier chromosomes have been identified in all prasinophyte genomes sequenced to date and this is an exciting development in researching immunity in these organisms. Two whole genome sequences have been reported for viruses (BpV-1 and BpV-2) that infect *Bathycoccus prasinos* [31] (Table 1). Unexpectedly, two long protein coding sequences (CDSs) were found within both the BpV-1 (11,202 and 17,067 bp) and BpV-2 (9,378 and 11,028 bp) viral genomes, which have no close taxonomic or functional matches in the public databases. These CDSs constitute between 10% and 15% of the entire genome and their presence raises the question as to what role they perform, particularly as genome reduction is a general feature of viruses.

*Bathycoccus* virus genomes appear to have acquired a heat shock protein *hsp70* gene from their host [31], the recruitment of which can greatly increase viral survival [59]. During infection, viruses can induce heat shock proteins resulting in enhanced viral infection, which has been demonstrated in detail for Hsp70. In eukaryotic cells, Hsp70 chaperone proteins are known to be involved in functions that support protein folding, translocation and assembly, and preventing apoptosis. In well-characterised viral infections, such as those of adenoviruses, these functions have been shown to aid maturation of viral proteins during infection and delay cell death enabling viral infection to reach completion. If BpVs can express their own Hsp70 proteins, rather than recruit host Hsp70 proteins, then they can regulate the expression of these proteins, and in the late stages of infection can suppress them to enable apoptosis and thus facilitate cell lysis.

Notably, the BpV genomes do not encode for certain amino acid biosynthesis pathway genes, such as a 3-dehydroquinate synthase gene, that are found in the genomes of prasinoviruses that infect *O. tauri* (OtVs), *O. lucimarinus* (OlVs) and *M. pusilla* (MpV-1) [31]. These genes encode for enzymes involved in the synthesis of essential amino acids that are formed in complex pathways when compared to the synthesis of nonessential amino acids. Their absence in these *Bathycoccus*-specific viruses is as yet unexplained. It has been postulated that the *Ostreococcus*-specific and *Micromonas*-specific dsDNA viruses encode certain hydrophilic and aromatic amino acids, as they do not utilise their host’s biosynthetic pathways for these important resources during infection. This may reflect differences in the host species protein metabolism or divergences in viral infection and replication strategies. A previous study isolated the OVs and MpV-1 from coastal lagoons but the BpVs were isolated from coastal open ocean seawater [31] and perhaps environmental factors/ecological niches play a role in the replication strategy of these particular viruses.

## 5. *Ostreococcus*: The Simplest Viral Host of Them All

The prasinophyte *Ostreococcus tauri* was first described in 1995, following isolation from a coastal lagoon in France [60,61]. The host genus *Ostreococcus* is emerging as a suitable model plant organism due to an array of attributions [26,27]. These include its cellular simplicity (haploid with just a single mitochondrion, chloroplast and Golgi body, no cell wall or motility apparatus e.g., flagella). *Ostreococcus*, unlike *Micromonas*, is not flagellated and unlike *Bathycoccus* does not have scales or liths on the outside of the cell); its phylogenetic positioning as an early-diverging green plant lineage [26,27]; ease of keeping in culture; successful genetic transformation [45]; availability of four complete fully annotated genomes [30,31,32,33]; complete mitochondrial and chloroplast genomes available for *O. tauri* [46] and proteome analysis [62,63]. *Ostreococcus* has emerged as an ideal model organism in studies of signalling in eukaryotic cells [64] and light and circadian clocks or oscillators [65,66,67]. The latter is a research field that endeavours to gain understanding about the in-built regulators of physiological responses of organisms to the 24 h daily cycle of changes in the Earth’s environment. *Ostreococcus* undergoes binary cell division and this can be manipulated by light/dark cycles in laboratory controlled conditions. *Ostreococcus* is a highly promising candidate for use as a model organism due to its simplicity as a eukaryote that can be used to extrapolate further for more complex eukaryotes. *Ostreococcus* could be used to study how cells respond to light, nutrient changes and to further investigate photosynthesis and cellular metabolism.

## 6. *Ostreococcus* and Its Viruses as a Host–Virus Model

With the early discovery of viruses that infect bacteria (bacteriophages), research focus was trained on a select number of model systems under laboratory conditions to characterise fully the dynamics of these viruses and their host interactions [68]. For eukaryotic virus systems, *Ostreococcus*-associated viruses form an equally attractive model candidate. The *Chlorella* algal host–virus system has become a classic model in algal virus research [12,69]. However, chlorovirus hosts are endosymbiotic zoochlorellae occurring within a range of hosts, including ciliates and metazoans, and chloroviruses are found in freshwater environments whereas OVs are representative of algal viruses occurring in marine environments.

The very first report of the existence of viruses targeting *Ostreococcus* species was in 2001, during monitoring of a picoplankton coastal bloom [70]. TEM analysis reveals *Ostreococcus*-specific viruses have similar morphology to other prasinoviruses, with capsid diameters ranging between 100–120 nm (Figure 1D–H). Assessments of the life cycle of OtV5 in culture revealed a latent period of 8 h, followed by cell lysis at 12–16 h post-inoculation [30]. Complete viral genomes were observed as early as two hours following inoculation. Host cell chromosomes remain intact throughout the infection cycle and are seen to decrease only as lysis occurs. TEM analysis confirmed viruses are localised to a region of the cytoplasm and do not associate with the nucleus or other organelles [30]. *Ostreococcus* and their viruses are globally distributed in coastal and open-ocean euphotic environments [34,36,43]. Viruses that infect *O. tauri* have been reported to be prevalent in coastal sites [32,33,36], whilst OlVs have been detected in more widespread marine locations, including oligotrophic sites in the Atlantic and Pacific Oceans [34,36,71,72], although this geographical distribution is not related to genetic distance, based on analysis of the *polB* gene [36]. Persistence of OVs and other prasinoviruses is linked to their environments. For example, MpVs are detected year-round, even in the absence of their hosts [53], and although the abundance of OtVs varies over time, they have also been detected throughout an annual sampling period [36]. To date, more than 300 OtVs have been sampled [36,56,71] and three complete OtV genomes [30,32,33], seven OlV genomes and one OmV genome sequenced and described, (with the latter being made publicly available—NCBI accession number NC_028092—but not yet published) [31,34] (Table 1) leading to insights into virus–host interactions, such as HGT events. The OtV-2 genome contains 42 unique genes with predicted functions not found in the genomes of the OtV-1 and OtV-5 viruses that infect high-light adapted hosts [33]. These include a putative cytochrome *b*_5_ gene, the function and structure of which have recently been characterised, that is located in the same region of the OtV-2 genome as putative RNA polymerase sigma factor and high-affinity phosphate transporter genes, all three of which appear to have been acquired via HGT from the eukaryotic host [33].

**Figure 2:**
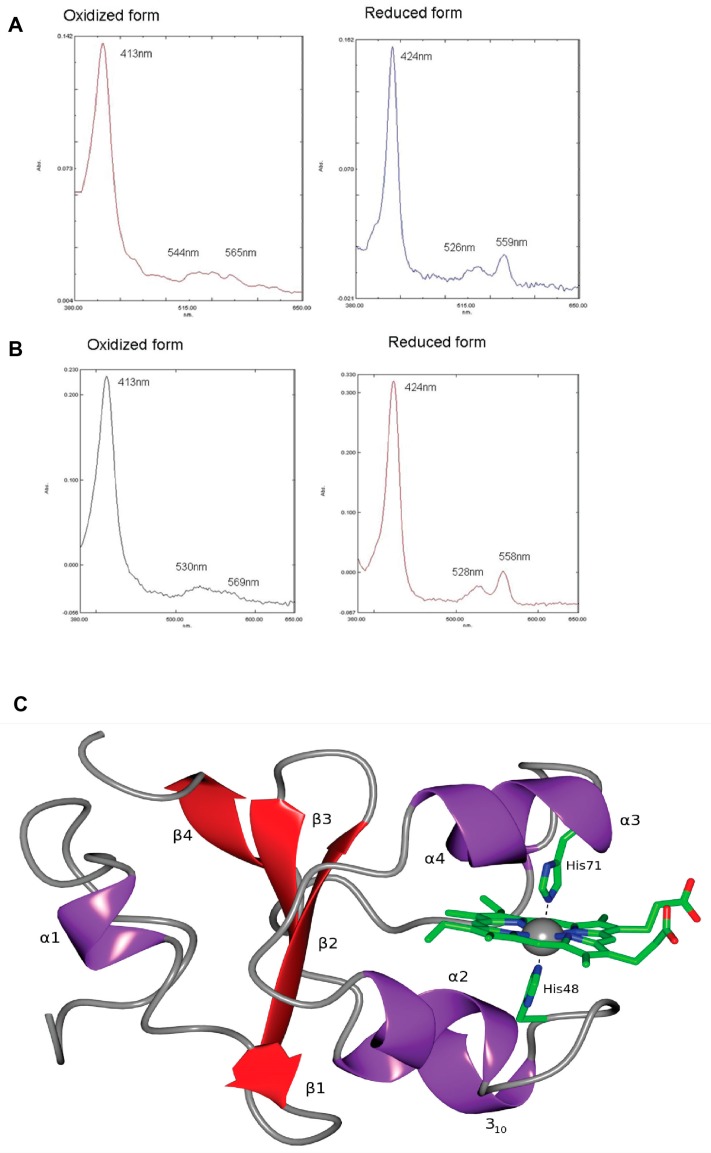
Characterisation of the OtV-2 virally encoded cytochrome *b*_5_ protein. Absorbance spectra for oxidised and reduced forms of (**A**) human cytochrome *b*_5_ protein and (**B**) OtV-2 viral cytochrome *b*_5_ protein and (**C**) structural display of the OtV-2 protein as a ribbon diagram. Adapted from [37].

Apart from members of the giant virus family the *Mimiviridae* [73,74], OtV-2 is the only other virus known to possess a putative cytochrome *b*_5_ gene [37]. Why the largest viruses ever to be described and a virus infecting the smallest known free-living eukaryote all putatively encode a cytosolic cytochrome *b*_5_ protein remains an unsolved mystery, as does the actual function of cytochrome *b*_5_ itself. Cytochrome *b*_5_ is an ubiquitous electron transport protein and can exist in two forms—a soluble enzyme used during photosynthesis in bacteria and a membrane-bound enzyme in animal tissues to reduce hemoglobin [75]. Cytochrome *b*_5_ exists typically as a membrane-bound protein inserted in the outer membrane of mitochondria and the endoplasmic reticulum via an alpha helix at its carboxy-terminus. However, the cytochrome *b*_5_ encoded by OtV-2 lacks this alpha helix and, as the hydrophobic C-terminal anchor is missing, the protein is not membrane-bound. The cytochrome *b*_5_ protein encoded by OtV-2 was cloned, biochemically characterised and crystallography was used to resolve its three-dimensional structure [37] (Figure 2). It was found that the absorption spectra of oxidised and reduced recombinant OtV-2 cytochrome *b*_5_ protein were almost identical to those of purified human cytochrome *b*_5_ (Figure 2A,B). The virally encoded cytochrome *b*_5_ was also substituted for yeast cytochrome *b*_5_ activity to confirm the viral cytochrome *b*_5_ was enzymatically active. Although structurally similar to other known cytochromes *b*_5_, the viral version, by lacking a hydrophobic C-terminal anchor, is the first cytosolic cytochrome *b*_5_ to be characterised (Figure 2C). The function of the viral version has seemingly diverged from that of its host protein to enable a different role during viral infection. The viral cytochrome *b*_5_ protein was seen to have a portion of the haem-binding domain missing when compared to the host version, indicating that the virus and host utilise cytochrome *b*_5_ for different functions. The viral protein lacks a reductase domain that confirms a divergence in function, although this physiological role remains unknown. As the host cell interior is ostensibly at the extremities of physical eukaryotic cell size, perhaps the occurrence of a virally encoded cytochrome *b*_5_ in the cytosol of the host cell enables electron transfer to occur more easily during the virus infection process?

## 7. General Features of Prasinovirus Genomes

There is a high degree of evidence of HGT in the prasinovirus genomes, including gene acquisitions from their prasinophyte hosts, as well as other eukaryotes and bacteria. There are some unique genes not found in other viruses and genes that share close homology to host genes, indicating lateral gene transfer events have occurred between host and virus in these systems. Novel genes encoded by prasinovirus genomes include a 3-dehydroquinate synthase, glycosyltransferases, *N*-myristoyltransferase, methyltransferases, 6-phosphofructokinase and prolyl 4-hydroxylase [31,32,33]. Further, the high number of eight major capsid proteins encoded by these viruses is unique [31,32,33] and could have implications for host interactions, for example viral adsorption to the cell membrane, although this area has yet to be explored.

Prasinovirus genomes are smaller than most other phycodnavirus genomes, ranging from 184 to 198 kbp (Table 1), compared, for example, to the coccolithoviruses, which can be as large as 415 kbp [12,76,77]. The smaller sizes of prasinovirus genomes can be attributed not only to fewer coding DNA sequences (CDSs) but also to a general trend towards smaller CDSs and intergenic regions. For example OtV-2 [33] contains a similar CDS number to the phycodnavirus *Heterosigma akashiwo* virus, HaV-53 [78], (237 versus 246 CDSs), but has a 110 kbp smaller genome (Table 1). The GC content of prasinovirus genomes is lower than that of their host genomes—37%–45% GC [31,33] compared to 50%–64% GC [27,28,29], respectively (Table 1).

To date, all sequenced dsDNA prasinovirus genomes have 125 predicted genes in common. A high degree of collinearity is observable between prasinovirus genomes, except for the very ends of the genomes, approximately 10,000 bp at either end, which are the sites of terminal inverted repeats [31]. There is a 32 kbp central inverted region in the genomes of two subgroups (termed type II viruses) of OlV genomes [34], indicating an intriguing evolutionary event in these viruses. Examining the conservation of prasinovirus genomes in comparison to their host genomes, it is worthy to note that the host genomes exhibit greater plasticity, have undergone greater evolutionary divergence in comparison to their associated viruses and there is lower nucleotide variation between virus genomes compared to their host genomes [31]. All members of the Mamiellales have outlier chromosomes with similar predicted gene functions but low sequence homology (11% versus 89% in non-outlier chromosomes between *O. tauri* and *O. lucimarinus*). The Mamiellales need to strike a balance between high genetic variability, short replication time and a rapid response to infection. This may be explained by the selection pressures exerted by viruses and the subsequent response of the host in changes in arrangement of the outlier chromosomes and differential expression rates in response to viral infection. This may also reflect the divergence within the *Ostreococcus* genus as it comprises several clades. In a study by Moreau and colleagues, the amino acid sequence identity of prasinoviruses and their respective host proteomes were compared [31]. The six prasinoviruses examined shared between 58% (OtV-1 and BpV-2) and 98% (OtV-1 and OtV5) average amino acid identity among their orthologous genes. This identity was consistently lower between the host’s orthologous proteins than between their viruses (*O. tauri* versus *O. lucimarinus* was 73.8% similarity compared to 81% between their viruses; 58.4% similarity between *O. tauri* and *Micromonas* sp. compared to 67.6% similarity between their viruses; and 54.8% similarity between *O. tauri* and *Bathycoccus* sp. compared to 58.5% similarity between their virus amino acid identity), indicating the evolutionary distance may be greater between hosts than their viruses. This observation was also reflected in the percentage of common genes between hosts being lower than between their viruses.

The level of divergence between hosts has implications for their cospeciation patterns with their respective viruses. A high degree of cospeciation indicates viruses are more likely to be highly specific to a particular host and less likely to switch between different hosts. A study of cospeciation between prasinoviruses and their Mamiellales hosts isolated from open ocean waters found that although a high degree of host specificity does exist, infection of different hosts species within a genus was also observed [79]. Cophylogenetic analysis revealed a likely complex coevolution of prasinoviruses and their hosts, with indications of host switching and varying susceptibility among host strains, potentially as a result of differing resistance [58,80], which may lead to reduced growth, as well as increasing susceptibility to other viral strains. It was postulated that cospeciation seen in this marine algal virus system was a result of close evolutionary ties, and therefore adaptation, between host and virus, as an open ocean system offers few physical barriers to dispersal with a higher potential for encountering a wide range of hosts, hypothetically affording more opportunities for host switching [79].

Chloroviruses are the prototype model for the *Phycodnaviridae* and 22 core orthologous genes are shared between prasinoviruses and chloroviruses, which range between 66% and 100% in similarity [34]. Of the 22 genes, 19 have a predicted function in DNA replication (nine genes) or protein (four), nucleic acid (four), sugar (one) and lipid (one) metabolism. The average identity of the core genes at nucleotide level was 88% between OlVs and 85% across all *Ostreococcus* viruses but dropped to 62% for prasinoviruses as a whole. Prasinoviruses and chloroviruses shared an average of 30% identity and were identified as the most closely related phylogenetically within the *Phycodnaviridae*. It may therefore be advantageous to build upon the knowledge acquired about chloroviruses by expanding the research conducted on their closest known relatives, the prasinoviruses.

## 8. Prasinovirus Gene Repertoire Contains Unique Highlights

Viruses can hijack a host-derived pathway and modify it in favour of virus metabolism. A key example of this is the acquisition of a coccolithophore host sphingolipid pathway by the coccolithovirus *Emiliania huxleyi* virus (EhV) [81] to enable a metabolic shift directing sphingolipid synthesis towards virus assembly and infectivity [82]. A number of genes not previously described in viruses are found in prasinovirus genomes. These include a cluster of genes found in the *Ostreococcus*- and *Micromonas*-specific prasinovirus genomes involved in the biosynthesis of amino acids [30,31,32,33]. The amino acids valine, leucine and isoleucine are synthesised using the acetolactate synthase gene, which is found in the MpV-1 and OV genomes and appears to have closest homology to bacterial genes but is not present in the BpVs, or in any other known virus [31,32,33]. Codon usage for leucine and valine are higher in all genomes compared to the BpV genomes but isoleucine is highest in MpV and lowest in OtV-1. The aromatic amino acids tyrosine, phenylalanine and tryptophan are synthesised with involvement of the enzyme 3-dehydroquinate synthase. A 3-dehydroquinate synthase gene has been identified only in OV genomes (with the exception of OtV-2 that infects the low-light ecotype of *Ostreococcus* [33]) and is unique among all viral genomes described to date. Prior to the availability of whole prasinovirus genomes, Mimivirus was the only virus known to encode an asparagine synthase gene [83]. This gene is also present in OVs and MpV-1, but not in BpV genomes [31,32,33], and was recently described in a virus that infects the prymnesiophyte *Phaeocystis globosa* (PgV) [84]. It has been postulated that most prasinoviral amino acid metabolic genes were acquired from bacteria, with the exception of asparagine synthase that instead appears to have originated from a eukaryotic source within the green plant lineage [31]. Interestingly, it would appear there has been no exchange of the asparagine synthase gene between virus families, or with their respective hosts, since both have seemingly undergone independent evolution [85]. The conversion of aspartate to asparagine occurs in cellular organisms across the tree of life but only in a small representative of two virus families, the *Phycodnaviridae* and the *Megaviridae*. An important question to pose is why such contrastingly different viruses have this particular basic housekeeping gene in common.

MpV-1 is the only prasinovirus, and in fact the only virus reported to date, that encodes for acetaldehyde dehydrogenase and oxovalerate aldolase enzymes [31], which are involved in the conversion of 4-hydroxypentanoate to acetaldehyde and pyruvate, and toxic aldehyde to harmless acetate in acetaldehyde metabolism, respectively. These enzymes perform important functions during oxidative stress and scavenge aldehyde in the cell that is produced during oxidative degradation of lipid membranes. The aldehyde dehydrogenase superfamily of enzymes is well represented in plants and these enzymes are commonly involved in stress response pathways. There are also conserved domains in the putative acetaldehyde dehydrogenase gene in MpV that are found in the TPP enzyme superfamily. Interestingly, these enzymes are involved in the biosynthesis of the amino acids isoleucine, leucine and valine. This adds further evidence pointing to the preferred use of certain amino acids by prasinoviruses indicating these may be important in capsid formation. It would appear prasinoviruses OVs and MpV favour the amino acid ratio towards these particular amino acids. These genes are also found clustered at the 5’ extremities of these viral genomes indicating their possible early transcription in the infection process.

Two distantly related forms of DNA ligase are encoded for by different members of the NCLDV group. All prasinoviruses have ATP dependent DNA ligases, not nicotinamide adenine dinucleotide(NAD)-dependent ligases. The evolution and acquisition of these genes in the NCLDVs is unclear, as phylogenetic analysis has indicated either form of DNA ligase may have appeared in these viruses first [24]. The prasinoviral genomes are linear with terminal repeat regions on their ends. Such repetitive regions are indicated to maintain genome stability [86] and it has been hypothesised that repetitive regions at the extremes of virus genomes were the precursors to telomeres in cellular chromosomes.

Viral replication of large DNA viruses requires a supply of deoxynucleotides. An essential intermediate in the synthesis of the deoxynucleotide dTTP is dUMP, which certain prasinoviruses produce using dCMP deaminase [31]. Deoxycytidine deaminase, thymidylate synthase, thymidine kinase and ribonucleotide reductase are all enzymes involved in dTTP synthesis. The dCMP deaminase enzyme is encoded by all *Chlorella* viruses [87] and is also found in OtV-2 [33], MpV-1, and OlV-1 [31], but not in any of the remaining prasinoviruses. Instead, they utilise ribonucleotide reductase in the pathway to synthesise dUMP for dTTP synthesis. Similar to chloroviruses, prasinoviruses encode for dUTPase and thymidylate synthase but only prasinoviruses also encode thymidine kinase, a key enzyme in the pyrimidine synthesis pathway involved in dTTP synthesis.

Unique to eukaryotes, mRNA capping is a post-transcriptional modification of messenger RNA (mRNA) that viruses can manipulate to ensure their mRNA is efficiently translated, whilst also gaining protection from cellular exonuclease degradation and recognition as foreign RNA by their hosts [88]. All prasinoviruses encode two mRNA capping enzymes, whilst *Chlorella* viruses encode one [31,33]. Methyltransferases can also offer a means for viral DNA to be protected from host cellular defence mechanisms. DNA methyltransferases are not commonly encoded by viruses but are found in bacteriophages and members of the *Phycodnaviridae*, namely chloroviruses [89], phaeoviruses [90] and prasinoviruses [31,33].

All prasinovirus genomes sequenced to date encode for as many as eight putative major capsid proteins (MCP) except the BpVs which lack MCP 1 maybe due to gene loss [30,31,32,33]. Most viruses encode for a single MCP and additional minor capsid proteins. The roles of these proteins may be related to structure and assembly but also to adsorption and host cell membrane fusion. Such a surprisingly high number of putative capsid proteins may indicate complexities or subtleties in the structure of the capsids of these viruses, although this has yet to be examined in depth. More complex viruses encode several versions of capsid proteins that are then used to build up the capsid structure surrounding the virus genome. Virus genome size is minimised by the formation of a capsid from multiple copies of a major capsid protein. So why do prasinoviruses encode multiple major capsid genes encoding for multiple capsid subunit proteins? The complexities of the capsid structure may hold functions pertaining to assembly, encapsidation, how entry into the host cell is executed and cues for adsorption and disassembly. TEM has shown OtV-1 capsid fuses with and remains fused to the host cell membrane [32]. Do any of the MCPs play a role in evading a host antiviral response? To begin to resolve the roles of these multiple MCPs in prasinoviruses, techniques such as X-ray crystallography and three-dimensional electron cryo-electron microscopy (cryo-EM) will need to be employed. TEM analysis has confirmed the prasinoviruses have an icosahedral structure (Figure 1). Icosahedrons typically have 12 vertices with 5-fold symmetry, and 20 triangular faces with 3-fold symmetry and 30 edges with 2-fold symmetry [77]. Much structural work has been conducted into viruses (as reviewed in [91]). As capsids are complex structures with hundreds of subunits, not all of the identical structures e.g., pentamers and hexamers, have been characterised in dsDNA virus families such as papillomaviruses and adenoviruses. Prasinoviruses must have very complex and intricate icosahedral architecture in their capsid structures. Considering the prasinoviral capsids are between 100 and 120 nm in diameter and interact within a host cell smaller than 1000 nm (meaning viruses are about one-eighth of the host cell size), their architecture, packaging and maturation must reflect this in the number of capsid proteins required. The burst size for prasinoviruses has been estimated to be less than 100 virions per cell and experimental data has indicated between six and 15 viruses are produced per host cell [30]. Considering that the host nucleus, mitochondrion and chloroplast remain intact throughout virus assembly in the cytoplasm [30], this adds to the global constraints on virus particle structure and the number of different capsid proteins required may reflect this. The internal pressure of packaging a genome inside the capsid will also influence the structure.

## 9. Viral Sugar Metabolism

Glycovirology is a newly emerging area of interest in virus–host interactions, studying how viruses manipulate the host glycome. The field of glycobiology is important as a potential focal point for antiviral therapy. It has been a point of interest that, like the chloroviruses, the prasinoviruses encode several glycosyltransferases, which are likely involved in glycosylation of viral components usually facilitated by the cellular glycosylation machinery. Sugar manipulation enzymes, such as 6-phosphofructokinase and glycosyltransferases, are encoded by prasinoviruses [31,32,33]. Glycosyltransferases (GTs) are known to be encoded by certain viruses including bacteriophages, phycodnaviruses, baculoviruses, poxviruses and herpesviruses (reviewed in [92]). GTs are instrumental in the formation of glycans. Viruses use glycans to ensure correct folding and conformation maintenance of viral glycoproteins and also for recognition for attachment to the host cell surface. MpV-1 and BpVs encode for three GTs [31], whilst OtV-2 and the remaining OV genomes contain four and six glycosyltransferase genes, respectively [31,33]. In the chloroviruses, GTs are postulated to be involved in post-translational modification and glycosylation of the capsid [92,93]. It has also been speculated that the chloroviral GT enzymes may have been acquired from a bacterial source and existed prior to formation of the cellular endoplasmic reticulum and Golgi body [93], as these organelles are not involved in chloroviral glycosylation. With the number of capsid proteins and glycosyltransferases encoded for by prasinoviruses, it would appear glycoconjugates are as vital for prasinovirus virion structure as they are in chloroviruses such as the prototype PBCV-1, which has six glycosylated sites on the MCP [93]. Of further note, is the fact that *N*-linked glycans are typically derived from asparagine and the OtVs encode asparagine synthase and, for example, OtV-1 encodes its own asparagine synthase and serine/threonine protein kinase [32].

An intriguing discovery was the presence of a gene, *pfk1*, encoding 6-phosphofructokinase (PFK), in both the MpV-1 and OV genomes [31,33], although not encoded for by the BpVs [31] or, in fact, by any other virus described to date. PFK is the key regulatory enzyme in the glycolysis metabolic pathway that converts glucose to pyruvate and ATP. PFK catalyses the glycolysis step that results in production of fructose-1,6-bisphosphate, which is not simply a metabolic intermediate but plays an important role in cell signalling and has been shown to delay cellular death in animal tissues [94]. The role of PFK in tumourigenic cells is a major focus for medical research at present. It has been shown that with increased PFK activity cell cycle progression is promoted and cell proliferation increases, whilst a decrease in PFK activity is linked to the onset of apoptosis [95]. The role of this crucial enzyme in cell metabolism could now be further revealed by studying the production of this virally encoded enzyme during infection of simple, minimalist algal host cells. Why does only this subset of prasinoviruses encode for such a centrally key metabolic enzyme? It seems plausible the viruses are exploiting the role of this enzyme in driving energy production by harnessing cell proliferation mechanisms to increase virus production, whilst also delaying apoptosis. It would be timely to widen the search for virally encoded PFK enzymes, as viral versions may remain undetected thus far and instead are amongst the countless hypotheticals and ORFans due to the divergence of viral from non-viral versions of the gene. It is also worth highlighting that PFK is the only glycolysis enzyme encoded by the OVs and MpV-1 and these viral genes share homology with bacterial genes, not equivalent host genes.

## 10. Host Resistance

Researchers have begun to explore viral infection in *Arabidopsis*, the classic terrestrial plant model organism, but not in a marine equivalent that is comparatively simple and known to be open to cellular manipulation e.g., *Ostreococcus*. In this regard, plant–virus coevolution requires established systems for in-depth analysis of how hosts react to infection e.g., development of resistance. It has been reported that *Ostreococcus* experiences high levels of infection by its associated viruses [36] and in response can develop resistance to viral attack [80], similar to that described in species such as *Prochlorococcus* [96]. Resistance to viral infection in *O. tauri* has been observed to be maintained for at least two years [58] and described in all three host genera [80] but can incur a cost, namely reduced growth rates and/or vulnerability to infection by other viruses. It has been proposed that the susceptibility of the *Ostreococcus* host to viral attack is related to the size of two outlier chromosomes [71], one big (BOC) and one small (SOC) similar to the theory purported to explain potential viral susceptibility in *Bathycoccus*.

In a recent transcriptional study of *O. tauri* in response to infection by OtV-5 in culture, the number of differentially expressed genes located on chromosome 19, the identified SOC, was markedly disproportionally higher than on other chromosomes [58]. The SOC has a bipartite structure being divided into an active left side during viral resistance and an active right side during susceptibility to infection. Most of the differentially transcribed genes located on the SOC in resistant hosts were associated with carbohydrate metabolism and transport. Four amino acid biosynthetic pathways were seen to be downregulated whereas post-translational modification and chaperones were upregulated. A transmembrane phosphate transporter was overexpressed and a calcium transporter underexpressed. Differential expression was seen in genes encoding translation, transcription, protein modification and turnover, amino acid transport and modification and other transporters. Viral immunity also appeared to be linked to a downturn in amino acid production. What is the cost of encoding two chromosomes involved in host defence? The cost to the cell may well be slowed host cell growth as ribosomal subunits were underexpressed in resistant cells. Histone modification genes were over expressed indicating likely chromatin restructuring occurred during resistance. By altering transporter activity, the host cell may be altering the available substrates for viral replication to occur.

Higher levels of GTs are located on the SOC and become overexpressed in resistant cells, whilst GTs are downregulated on the BOC. The SOC genes with known function encode methyltransferases, glycosylation-related genes and membrane proteins. Interestingly, both OtV genomes and the SOC in *O. tauri* encode a NAD-dependent epimerase/dehydratase with the host gene bearing closest homology to a gene in higher plants whilst the viral gene is closest to a bacterial gene. 

A FkbM methyltransferase gene is present in both viral and host genomes, occurring close to the sugar gene cluster in the SOC, and is predicted to play a role in glycan methylation [32,58]. Glycans are carbohydrate modifications on proteins or lipids that act as ligands that bind carbohydrate-binding proteins called lectins. Glycans are the key at the interface of virus and host. Viruses use their own or their host glycans for replication and infectivity. A virus capsid possessing glycans can act as a ligand for host lectins and host glycoconjugates that can in turn act as receptors for viral lectins and facilitate cell entry. It would appear *O. tauri* encodes its own versions of sugar enzymes and methyltransferases on the SOC that are replicated in response to initial infection events. The resulting events divert virus adsorption and result in glycosylation and methylation to provide protection away from the virus-directed pathways. The host is playing the virus at its own game and, by using different versions of the same gene, the host machinery is regaining control and blocking the virus from the point of adsorption. It has been postulated the OtV5 receptor may only be available at a certain point in the host cell life cycle. However, if the compact intracellular organisation and limited physical space available within the host cell is taken into account, then it would seem more likely the host will try to arrest infection earlier rather than later, so therefore, the point of viral attachment and adsorption would be a more logical stage to create resistance. Do the host flood cell surface receptors with host versions of sugars or does it alter the substrate pool, or perhaps both strategies are adapted simultaneously?

A long inverted repeat region (LIRR) was identified in the SOC and contains all the over-transcribed genes in the resistant cells and is the region that is silenced in the susceptible wild-type state [58]. In the LIRR, clusters of genes involved in carbohydrate metabolism were seen. The *O. tauri* SOC is unique by encoding a rhaman synthesis gene *RgpF* not found in any other eukaryote but only in bacteria. Rhaman synthesis performed by genes located on the SOC seems to be a key weapon in the host arsenal against viral infection. Notably, OtV-1 encodes a dTDP-d-glucose-4,6-dehydratase that shares closest homology with the metazoan *Nematostella*. This protein is also known as rhamnose synthase and is also encoded by the host. OtV5 encodes a GDP-D-mannose 4,6-dehydratase with closest homology to a gene in the chlorovirus PBCV-1 that is involved in rhamnose and fucose synthesis, which are monosaccharides commonly seen in the virion capsid but occur rarely in the host. This must be one of the key points that resistant hosts interfere with. Do prasinoviruses within an already exposed culture attach to host cell surfaces and employ host glycoconjugates to enable entry into the cell but the SOC switches and changes the glycobiology and available conjugates, so as to prevent viral infection? It would appear that the underlying mechanism here is related to glycan-mediated host–virus interactions. 

The SOC and BOC are able to swap genetic material between each other and rearrange within themselves, with transposons most likely playing a key role in this activity [58]. Karyotypic changes were observed in the size of the SOC in all resistant lines of host cells via duplications, deletions and possible translocations [58]. Are these changes causative or resultant of resistance? The glycobiology aspects of prasinophyte host–virus interactions represent an exciting area for future research to expand into. The next logical step is to take the analysis of chromosome 19, the SOC, into the natural environment.

## 11. Nitrogen Metabolism in Prasinophytes and Their Viruses

*Ostreococcus* and *Micromonas* contain genes for nitrate/nitrite transporters and reductases and *Ostreococcus* also encodes proteins involved in molybdate transport and metabolism [27,28]. *O. tauri*, *O. lucimarinus* and *B. prasinos* encode animal-type nitric oxide (NO) synthase (NOS) enzymes that appear to have been acquired via HGT [97]. This is of interest as NOS is a haem protein similar to cytochrome P450 reductase, and contains a haem domain, as does cytochrome *b*_5_. Interestingly, the NOS unveiled in *O. tauri* shares 45% similarity to human NOS and displays similar protein folding to the human version [98]. This was the first report of a NOS in a plant. The generation of NO in *O. tauri* was seen to be higher under high light irradiance and during exponential growth [98]. The production of NO is seen to increase with high light intensity irradiation, which may reflect clade assignation and ecotype niche partitioning and hence indicates a link between physiology and NOS activity. It would be interesting to explore such metabolic processes further, particularly in regard to active viral infection. It is unique in plants that *O. tauri* encodes NOS with similarities to animal and bacterial NOS [98]. Interestingly, nitric oxide has been implicated to have a role in immune responses to viruses in animals [99]. However, to date no research has been conducted into the role of these enzymes in algal host defence against viruses. Nitrogen, along with elements such as phosphorus, is an important limiting factor in viral infection [100].

## 12. Inteins in Prasinoviruses

Complete large inteins containing a homing endonuclease have been detected in the DNA polymerase gene of a number of prasinoviruses, including in the OtV-1 genome and other environmental *O. tauri* sequences (isolates OtV06_1, OtV09_561, OtV09_600) and BpV-2 [32,101]. Inteins are genetic selfish elements that insert themselves into conserved regions of conserved genes and are capable of self-splicing following translation [102]. The homing endonuclease facilitates the lateral transfer of their own coding region and flanking sequences between genomes and this process is called ‘homing’ and is recombination-dependent. It has also been reported that a recombination event occurred between two inteins found in OtV viruses infecting *O. lucimarinus* and *O. tauri* [101]. This finding could have important ecological and evolutionary significance and result in increasing virus diversity, as recombination events occur via this mechanism. Inteins have been adopted as biotechnological tools by exploiting their natural splicing mechanisms and their use in aiding protein purification [102]. Inteins are seen to be widely distributed in nature although their distribution is sporadic indicating lateral transfer events are at play. Recombination events between inteins found in OVs have been reported [101]. Prasinoviruses can seemingly co-infect the same host enabling lateral transfer of inteins between viruses. If prasinoviruses are similar enough genetically then they can coinfect the same host cell, which not only enables lateral transfer of inteins but has implications for the host too, in terms of being assaulted by more than one virus simultaneously. It has been postulated that co-infection is likely widespread and virus–virus interactions are common [102]; although most work has been conducted into bacteriophages, this may well also apply to eukaryotic viruses. If coinfection by two or more viruses can occur in a small host cell such as *Ostreococcus* then this has some major implications for viral evolution, viral exchange and infection dynamics. Clerissi and co-workers speculated that the higher concentration of prasinophyte hosts in lagoon environments may increase the incidence of inteins and their transfer, compared to similar events occurring in coastal and open waters [101].

## 13. Conclusions and Future Directions

It is now timely to establish the smallest known free-living eukaryotes at the base of the green algal lineage, particularly *Ostreococcus*, and their associated viruses as a suitable model system to examine in-depth the dynamics of marine algal virus infections. There exist large gaps in our knowledge surrounding viral life history and interactions with their hosts. Viruses that infect the prasinophytes can provide novel and incisive insights into how such model systems work and allow us to study processes including primary production, cellular resource allocation, genetic transfer events and evolution. Recent unveiling of the genetic treasures of the prasinoviral genomes and infection strategies, and those of their host responses to these remarkable features, are helping to open a whole new aspect to not only viral–host interactions but also cellular biology, genetics and physiology.

## Figures and Tables

**Table 1:** Key features of characterised prasinophytes and their prasinoviruses.

Host Species	Host Genome Size	Host GC Content (%)	Number of Chromosomes	Number of Host Genes	Virus Genome Size	Virus Genome GC %	Number of Viral ORFs	Average Viral ORF Length (bp)	Viral tRNAs
*O. tauri* clade C	12.6 Mb	59	20	8116	OtV1 = 191,761 bp	OtV1 = 45	232	750	OtV1 = 4
					OtV5 = 186,713 bp	OtV5 = 45			OtV5 = 5
*Ostreococcus* sp. RCC809 clade B	13.3 Mb	60	20	7492	OtV2 = 184,409 bp	OtV2 = 42.15	237	725	OtV2 = 4
*O. lucimarinus* clade A	13.2 Mb	60	21	7805	OlV1 = 194,022 bp	OlV1 = 41	268	732	OlV1 = 5
*O. mediterraneus* clade D	-	-	-	-	OmV1 = 193,301	OmV1 = 44.6	251	730	OmV1 = 5
*Micromonas* sp. RCC299	21.96 Mb	64	17	10,286	MpV1 = 184,095 bp	MpV1 = 39	203	793	MpV1 = 6
*B. prasinos* RCC 1105	151 Mb	48	19	7847	BpV1 = 198,519 bp	BpV1 = 37	210	746	BpV1 = 4
					BpV2 = 187,069 bp	BpV2 = 38	244	715	BpV2 = 4

*Bathycoccus* species virus had large predicted protein removed for the purpose of this table. The gene comprises 14% of the total genome. ORFs, open reading frames; tRNA, transfer RNA; bp, base pair; OlV, *Ostreococcus lucimarinus* virus; MpV, *Micromonas pusilla* virus; BpV, *Bathycoccus prasinos* virus.
